# Primary Oral Syphilis Presenting With Tongue Ulcers: A Case Report

**DOI:** 10.1155/crid/6388059

**Published:** 2026-09-09

**Authors:** Mazin Barry

**Affiliations:** ^1^ Infectious Disease Unit, Department of Internal Medicine, College of Medicine, King Saud University, Riyadh, Saudi Arabia, ksu.edu.sa; ^2^ Division of Infectious Diseases, Faculty of Medicine, University of Ottawa, Ottawa, Canada, uottawa.ca

**Keywords:** case report, oral syphilis, primary syphilis, tongue ulcers

## Abstract

Syphilis has historically been called “The Great Mimicker” because of its various stages and clinical signs. It typically occurs in one of three stages: primary, secondary, or tertiary. Primary syphilis often shows painless genital ulcers (syphilitic chancre), which can be hard to detect in women or men who have sex with men (MSM). Oral presentation of syphilis is extremely rare and can be a diagnostic challenge. Here, we present a rare case of primary oral syphilis in a healthy young MSM presenting with painless tongue ulcers for 7 days, 6 weeks after an oral sexual encounter. His rapid plasma reagin (RPR) titer was 1:16, and his *Treponema pallidum* hemagglutination assay (TPHA) titer was 1:10240. He was treated with benzathine penicillin G 2.4 million units intramuscularly once. Six weeks later, his symptoms completely resolved, and at 1‐year follow‐up he remained well with no recurrence, and his RPR titer dropped fourfold. The case highlights the diagnostic challenge of such a rare presentation of syphilis. Detailed sexual history in similar presenting patients is crucial to help include syphilis within the differential diagnosis.

## 1. Introduction

Syphilis is an ancient disease caused by infection with the spirochete bacterium *Treponema pallidum* subspecies *pallidum*. It is a major global health problem, and the incidence has been increasing, especially among men who have sex with men (MSM) [1]. Syphilis has three different stages: primary, secondary, and tertiary. Infection is transmitted during the first two stages when in direct contact with a sore or rash of a sexual partner. The primary lesion is usually a painless ulcer (syphilitic chancre), which often appears at the site of inoculation, mainly the external genitalia, vagina, anus, and rectum. These lesions are usually missed, especially in women and MSM [2]. Oral presentations are unusual; however, they have been increasing among MSM [3]. Although rare, lesions may affect any part of the oral cavity, including upper and lower lips, soft and hard palates, buccal mucosa, tonsil, uvula, lingual frenulum, gums, floor of mouth, and tongue. Here, we present a rare case of primary oral syphilis presenting with multiple tongue ulcers.

## 2. Case Presentation

A previously healthy 31‐year‐old man presented in 2025 to the Infectious Diseases (ID) outpatient clinic in Riyadh, Kingdom of Saudi Arabia (KSA), with tongue ulcers for the past 7 days. The ulcers were painless and did not affect his ability to eat or speak. He had never had similar ulcers on his tongue or in his mouth before, nor any previous lip lesions. He had no rashes or other lesions elsewhere on his body and reported no fever, headache, or systemic symptoms. Six weeks prior to his presentation, he engaged in oral fellatio with a new male partner without barrier protection, and his partner′s semen was ejaculated into his mouth. He had no prior sexual encounters before that incident, and no previous diagnosis of any sexually transmitted infections (STIs) or any testing for them. He was unaware of whether his partner had any STIs. He had no history of trauma to the tongue, recurrent aphthae, systemic symptoms including fever, loss of appetite, weight loss, or malaise. He had no cough, skin rash, sore throat, loose teeth, joint pain or swelling, genital ulcers, ocular symptoms, medication exposure, immunosuppression, or gastrointestinal symptoms. In addition, he had no known exposure to a patient with tuberculosis (TB), no raw milk ingestion, no family history of rheumatological conditions, inflammatory bowel disease (IBD), or malignancies. He does not smoke tobacco and does not consume alcohol.

**Figure 1:**
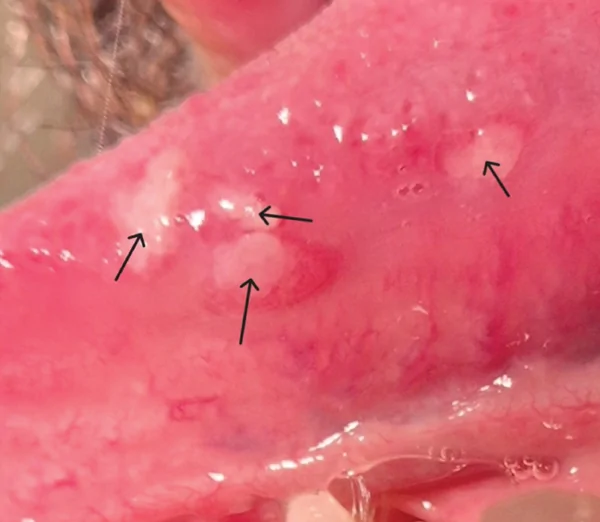
Lateral left side of the patient′s tongue with four superficial ulcers with irregular borders (arrowheads).

On physical examination, he appeared generally well with normal vital signs. The oral cavity exam revealed multiple small ulcers on the lateral left side of his tongue (Figure 1). There were four distinct lesions, less than 1 cm in size, less than 1 mm in depth, with well‐marked irregular borders, clear bases, surrounding erythema, no tenderness or bleeding, no exudate, and indurated on palpation. The remainder of the oral exam was normal, with no angular stomatitis. Neck examination revealed two small, palpable left cervical lymph nodes that were soft, mobile, nontender, and less than 1 cm in size. The rest of the physical exam, including genital and rectal examinations, was normal. Given the painless tongue ulcers and the recent sexual encounter, primary syphilis of the oral cavity was suspected clinically. An enzyme immunoassay (EIA) for total treponemal (TP) antibodies, IgM, and IgG was reactive; his syphilis rapid plasma reagin (RPR) titer was 1:16, and his *Treponema pallidum* hemagglutination assay (TPHA) titer was 1:10240. HIV antigen/antibody test was negative, and herpes simplex virus 1 and 2 (HSV‐1/2) IgG and IgM were negative. An oropharyngeal swab and urine sample were obtained for nucleic acid amplification test (NAAT) for *Neisseria gonorrhoeae* and *Chlamydia trachomatis*; both were undetected. Hepatitis B and C serologies were negative. His CBC, ESR, CRP, and metabolic profile were all normal. His chest x‐ray (CXR) was normal with no granulomas or fibrosis. The patient did not wish to undergo an invasive procedure like a biopsy; therefore, no direct lesion testing was performed. However, a provisional diagnosis of primary oral syphilis was made, and he was prescribed benzathine penicillin G 2.4 million units intramuscularly once. The patient tolerated the treatment well, with no reported adverse events. The Public Health Agency provided partner notification and counseling. Six months later, he returned for follow‐up with complete resolution of the tongue lesions, and his repeated RPR was 1:8. One year later, he remained well with no recurrence of any ulcers, and his repeated RPR was 1:4. Repeated HIV serology was also negative. He was scheduled for follow‐up in another 6 months. From the patient′s own perspective, he was satisfied with the diagnosis and outcome.

## 3. Discussion

The current case presented with a rare form of primary oral syphilis in the form of painless tongue ulcers. The differential diagnosis of tongue ulcers includes traumatic ulcers, aphthous ulcers, oral squamous cell carcinoma (OSCC), TB, deep fungal infections, Behçet disease, and HSV infection. His symptoms were present for the past 7 days before presentation. He had no trauma to the tongue, no previous similar episodes, no risk factors for OSCC like smoking or alcohol use, no known TB exposure or evidence of old TB on the CXR, no joint or ocular symptoms, no skin rash, and no family history of Behçet disease or IBD. However, the sexual activity was of high risk for acquiring an STI, including syphilis, due to a lack of any barrier protection. His lesions developed 5 weeks after the sexual encounter, which is within the incubation period of primary syphilis, which ranges between 9 and 90 days [4]. Hence, a provisional diagnosis of primary oral syphilis was suspected. In the absence of a biopsy, the presumptive diagnosis was based primarily on serology, using both TP and nontreponemal (NTP) antibodies, with reactive TPHA and RPR, respectively.

Oral syphilis was first documented in a dental report in 1904, showing that 32% of extragenital syphilis cases involved the oral cavity [5]. More recently, a systematic review of 145 cases with oral manifestations of early syphilis described two distinct clinical phenotypes of oral syphilis: ulcerative or erosive lesions with mucous patches, and the tongue was the most affected site at 42.8%. Additionally, 60.7% of cases involved only one anatomical site, with ulcerative lesions being the most common at 65.9%. Furthermore, these cases were more likely to be diagnosed as primary syphilis [6]. Single painless lesions have also been reported [7]. The current case presented with a single anatomical site, and the most commonly reported involved site of oral syphilis with multiple lesions on his tongue. Table 1 demonstrates the number of cases with single anatomical involvement of the tongue reported in the literature.

**Table 1:** Cases reported in medical literature with single anatomical involvement of the tongue, with types of lesions and gender.

Number (*n*) of cases with a single anatomical location involving the tongue	Type of lesion, *n*(%): ulcer	Type of lesion, *n*(%): nodule	Type of lesion, *n*(%): mucous patch	Gender: male, *n*(%):	Gender: female, *n*(%)
33	21 (63.6)	3 (27.3)	9 (9.1)	24 (72.7)	9 (27.3)

In a systematic review of STIs manifestations in the oral cavity, primary syphilis presented frequently with ulcers, most frequently of the tongue, pointing out that oral syphilis has been on the rise, affecting people aged 20–39 years more commonly, which highlights the importance of interventions for preventive measures [8]. STIs in the oral cavity have increased due to more oral sex activities, such as cunnilingus, anilingus, and fellatio [9]. A cross‐sectional study among 2322 MSM in Australia found that these activities were significant risk factors for STIs [10]. The risk of transmitting syphilis during oral sex is estimated to be 1% per activity, especially if oral ulcers or genital sores are present during the activity [11]. The current case engaged in receptive oral sex (fellatio) without a barrier method, which increased the risk of acquiring syphilis.

Serological testing is the primary diagnostic method for syphilis, utilizing both NTP and TP antibodies. For primary syphilis, the NTP test RPR is reported to have a sensitivity of 86% and a specificity of 98%. The TP test TPHA has a sensitivity of 86% and a specificity of 96% [12]; however, the latter test cannot distinguish between past and current infection; therefore, both tests are typically performed in a two‐step process. Although no direct lesion testing was performed, the current case′s serological tests helped establish the diagnosis of primary oral syphilis. The absence of other signs and symptoms besides the tongue ulcers, along with the development of these lesions within 5 weeks of sexual exposure, further supports the diagnosis of primary oral syphilis, in addition to the resolution of symptoms and reduction of RPR upon follow‐up.

The combination of compatible exposure, incubation period, reactive TP serology, and RPR titer supported a diagnosis of primary oral syphilis, and the tongue ulcers were clinically suspected to represent the primary lesion. However, the absence of direct lesion testing limits diagnostic certainty.

## 4. Conclusion

The rare presentation of primary syphilis with tongue ulcers can pose a diagnostic challenge. Although direct confirmation was unavailable, this case highlights the importance of considering syphilis in the differential diagnosis of painless oral ulcers. A detailed review of sexual history can help guide the diagnosis. Healthcare providers and dental professionals should be aware of such uncommon presentations and remain highly vigilant in similar cases.

## Author Contributions

**Mazin Barry:** conceptualization, methodology, software, validation, formal analysis, investigation, resources, writing – original draft, writing – review and editing, visualization, supervision, project administration.

## Funding

No funding was received for this manuscript.

## Disclosure

The author have read and approved the final version of the manuscript. The corresponding author had full access to all of the data in this study and takes complete responsibility for the integrity of the data and the accuracy of the data analysis.

## Ethics Statement

This case report was approved by the Institutional Review Board, King Saud University, Ethical Approval Number: E‐25‐9887.

## Consent

Written informed consent was obtained from the patient for publication of this case report and accompanying images. A copy of the written consent is available for review by the editor in chief of this journal on request.

## Conflicts of Interest

The author declares no conflicts of interest.

## Supporting information


**Supporting Information** Additional supporting information can be found online in the Supporting Information section. Supporting Information.

## Data Availability

Data sharing is not applicable to this article as no datasets were generated or analyzed during the current study.
